# Quantitative Assessment of Preventive Behaviors in France during the Fukushima Nuclear Crisis

**DOI:** 10.1371/journal.pone.0058385

**Published:** 2013-03-07

**Authors:** Pascal Crépey, Mathilde Pivette, Avner Bar-Hen

**Affiliations:** 1 EHESP Rennes, Sorbonne Paris Cité, Paris, France; 2 Aix-Marseille Univ, UMR EPV Emergence des Pathologies Virales –190, Marseille, France; 3 Université René Descartes-Paris5, MAP5, Paris, France; Kagoshima University Graduate School of Medical and Dental Sciences, Japan

## Abstract

**Background:**

The Fukushima nuclear disaster has generated worldwide concern on the risk of exposure to nuclear radiations. In Europe, health authorities had to issue statements about the lack of usefulness of iodine based preventive treatments within their borders. However a lack of confidence in official messages has developed in various European countries due to recent perceived failures in managing public health crises. The lay population preventive behaviors in this context are largely unknown. Consequently, to examine the effects of public health crisis on lay behaviors leading to pharmaceuticals purchases, we studied the sales of iodine-based products in France before, during and after the crisis.

**Methods:**

We focused our study on 58 iodine-based drugs available with and without a physician prescription. Our data came from a stratified sample of 3004 pharmacies in metropolitan France. Our study period was from January 2010 to April 2012, with a focus on March-April 2011. We differentiated sales of drugs prescribed by physicians from sales of drugs obtained without a prescription. We used a CUSUM method to detect abnormal increases in sales activity and cross-correlations to assess shifts in sales timing.

**Results:**

Sales of iodine-based nutritional complements, and later sales of iodine-based homeopathic remedies, substantially increased (up to 3-fold) during a period of 20 days. Their temporal patterns were correlated to specific events during the crisis. Prescriptions for iodine-based homeopathy increased (up to 35% of all sales). Iodine pills, strictly regulated by health authorities, have also been sold but on a very small scale.

**Conclusion:**

These results indicate uncontrolled preventive behaviors resulting in the potentially unjustifiable consumption of available drugs. They have implications in public policy, and demonstrate the usefulness of drug sales surveillance for instantaneous evaluation of population behavior during a global crisis.

## Introduction

On March 11, 2011, a magnitude 9 earthquake followed by a 15-m tsunami wave damaged the Fukushima Daiichi Nuclear Power Plant (NPP) in Japan [1]. The disaster provoked the meltdown of three reactors leading to a level-7 nuclear incident [2]. In the aftermath, Japan had to face the worst nuclear disaster since the destruction of Chernobyl’s reactor number four in 1986. Nuclear disasters have global consequences, and populations are now highly aware of the potential public health consequences of radioactive discharges [3], [4].

In France, a severe loss of confidence in political authorities has been observed in matters concerning nuclear risk following the Chernobyl event. A study on a sample of the general population in France by Carlé and colleagues [4] showed that 75% of the respondents believed they had not been told the truth about the Chernobyl fallout. The same study showed that neither government representatives nor journalists were considered by a large majority of respondents to be technically competent and likely to tell the truth about nuclear issues. In addition, 68% of the respondents had no confidence in the abilities of the authorities to protect them in case of a major nuclear disaster.

This lack of confidence in health authorities was also observed during the last influenza pandemic. However, the reluctance to comply with official recommendations seems to be more related to differences in risk perception [5], [6], which resulted in a lower than usual pandemic influenza vaccination uptake [7].

During the Fukushima crisis, contrary to multiple unofficial and nonmedical sources, all French official communications indicated that using iodine was not useful as a preventive measure in France [8]. Potassium iodide (KI) pills are only used in case of risk of direct exposure to radioactive iodine isotopes to saturate the thyroid glands with nonradioactive iodine. In France, KI pills are army-regulated and only available in military controlled pharmacies or in pharmacies located in a 10-km radius from one of the 19 French NPPs [9]. However, several drugs, such as antiseptics, nutritional supplements and homeopathic remedies, containing relatively low concentrations of iodine, can be purchased in pharmacies with or without prescriptions.

Our study focuses on sales of the previously described drugs, on their timing, and on correlations with the timing of events that may have impacted those sales. Implications in terms of public health campaigns are discussed.

## Materials and Methods

We studied the changes in pharmaceutical sales of iodine-based drugs in France through a stratified random sample of 3004 pharmacies set up by the company Celtipharm [10]. Through their participation in the Xpr-So© system, the pharmacies have automatically reported their sales several times a day since 2007. Thanks to a regularly updated exhaustive database of the 22,458 active French pharmacies, a stratification to improve representativeness is performed on sales revenue (6 levels for global revenue and 4 levels per type of sales: prescribed drugs, OTC, and other type of sales), localization (5 geographic areas) and sales area (5 types, from rural to densely urban). Each stratum has a minimum of 30 pharmacies or is merged with neighboring strata. Sampling rates per strata are computed with the Neyman optimal allocation algorithm [11]. Extrapolations from the sample have been validated with data from drug manufacturers who distribute their products directly (and only) to pharmacies. We study sales data from January 2010 to April 2012 in order to include a reference period (the year 2010) and the period surrounding the Fukushima crisis. We compiled a list of 92 products containing or claiming to contain iodine based on a search for the pattern “iod*” in their name or principal ingredients. From that list, a total of 58 had been sold at least once in pharmacies from our sample during the study periods. We analyzed separately sales made with a physician prescription and those made without prescriptions. Sales were observed at the national and regional levels. The number of pharmacies selling the listed products was also analyzed. The list of products is given in Table S1. We computed cross-correlations of sales time series to quantify potential lags between types of sales. To detect unexpected increases in sales and assess durations of this kind of event we used a negative binomial CUSUM algorithm. We parameterized the CUSUM to compute its score from the difference between the cumulative sum of the sequence of observations and the maximum of variations observed the preceding month. We used a lag of a few days between the current observation and the preceding month to avoid tainting the “variation baseline” with the observed event. The same method, developed for real-time detection of a parameter’s deviation, is also classically used to detect disease outbreaks [12], [13]. Positive CUSUM score can occur during a normal year; consequently, to maximize the specificity of our indicator we set the detection threshold to the maximum value of the score obtained during an *a priori* “normal” year, i.e. the year 2010, preceding the Fukushima crisis.

## Results

**Figure 1:**
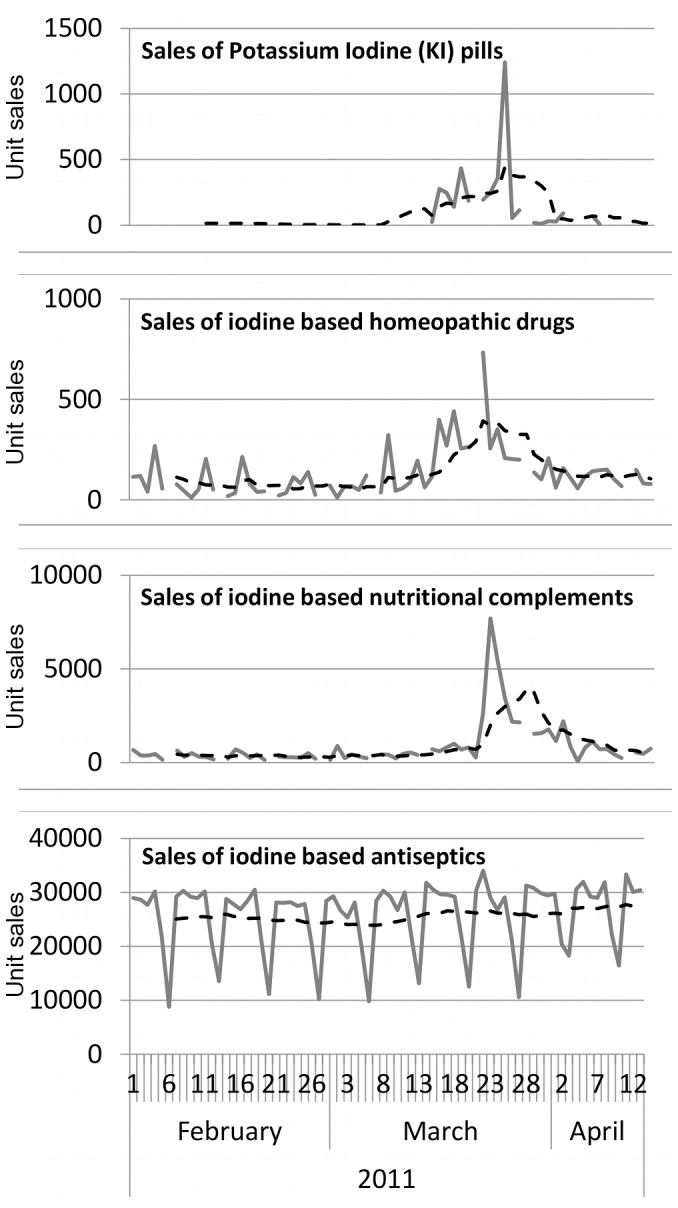
Sales of iodine based products (per units) in French pharmacies between February and April 2011. Dashed lines show the weekly sliding average.

**Figure 2:**
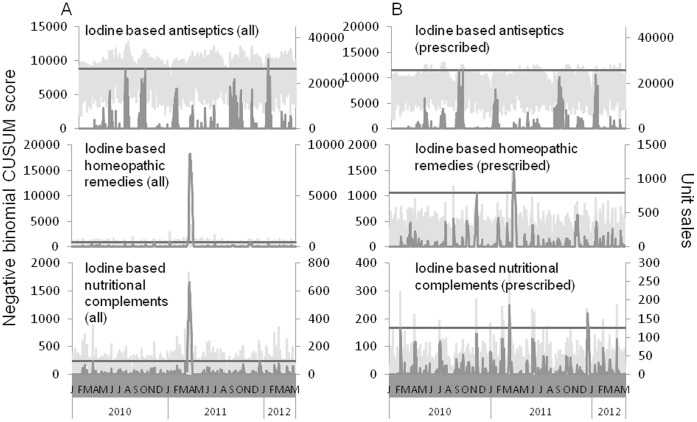
Dark gray lines stand for negative binomial CUSUM score for unit sales of Iodine based antiseptics, homeopathic remedies and nutritional complements (from top to bottom). The straight lines on the plots illustrate the threshold for the CUSUM score, defined as the maximum value of the CUSUM score in 2010. An increase in the CUSUM score indicates a strong positive deviation from the normal trend. Light gray lines represent unit sales volumes. Panel A shows results for all type of sales while panel B only shows sales with a physician prescription.

We defined four categories of iodine-based products: antiseptics, nutritional complements, homeopathic remedies, and KI pills. Figure 1 shows increases in sales in all product categories except iodine-based antiseptics during the period of the Fukushima crisis (February to mid-April 2011). In addition, although KI pills were only available to people living in a 10 km-radius from a NPP and were not released to the rest of the population for preventive purpose during the period, sales were registered in our sample specifically during the crisis. To confirm these increases, we computed a negative binomial CUSUM score [12] on the sales time series (Figure 2). We define the threshold for “sales outbreak” detection as the maximum CUSUM score obtained for the year 2010. Antiseptics (prescribed or not) do not cross the threshold in 2011, while iodine-based homeopathic remedies cross it during 22 days for all sales and 7 days for prescribed sales. Lastly, nutritional complements cross it during 19 days for all type of sales. Prescribed nutritional complements also slightly cross the threshold in 2011 but a few days prior to the Fukushima crisis. Sales of KI pills were almost nil outside of the Fukushima period making the CUSUM computation irrelevant. Comparisons between sales in 2010 and 2011 are given in Table 1. In particular, we notice a 144% increase of iodine-based nutritional complement sales in March 2011, but the largest sales increase concerns iodine-based homeopathic remedies, with a threefold increase compared to March 2010. We assessed the scale of the phenomenon by analyzing the number of pharmacies per day selling the identified products. In 2010, on a given day only 210 pharmacies on average sold iodine-based homeopathic remedies. During the 1-month period starting on March 11^th^, this number rose to 740 selling pharmacies per day on average and to 2,046 between the 21^st^ and 25^th^ of March 2011, representing 10% of the total number of stores.

**Table 1:** Comparison of iodine based products in one month in 2010 and the same month in 2011 after the beginning of the Fukushima crisis.

Estimated sales in French pharmacies (in units)	From March 11 toApril 11 2010	From March 11 toApril 11 2011	Percentage ofincrease
**Iodine based nutritional complement**	2545	6219	144%
**Iodine based homeopathic remedies**	10747	43912	309%
**Iodine based antiseptic**	758436	825963	9%

**Figure 3:**
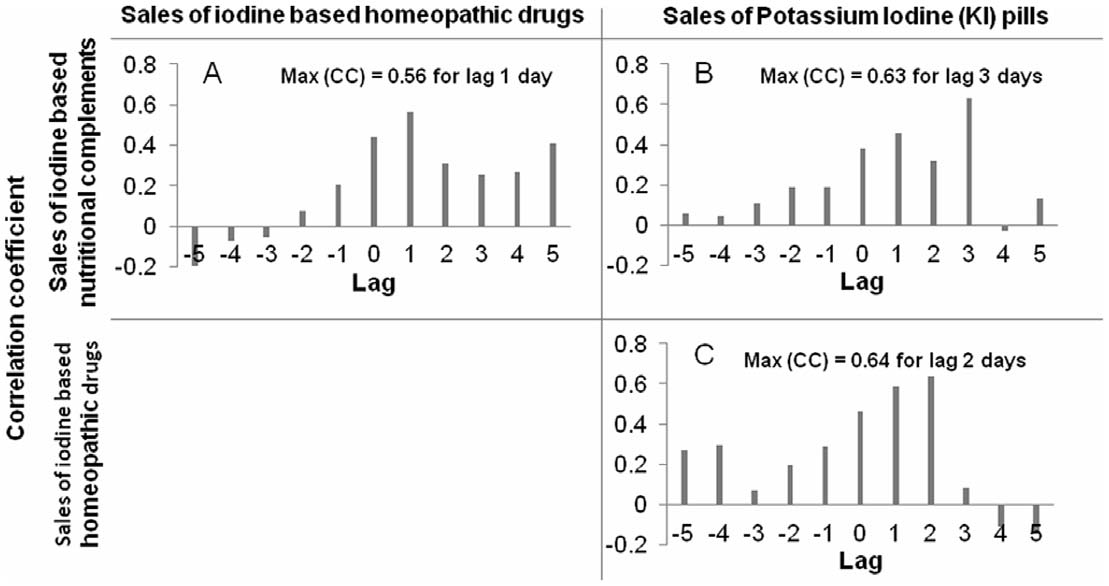
Cross-correlograms of sales of iodine based nutritional complements (INC), homeopathic drugs (H), and Potassium Iodide (KI) pills with lags from −5 to +5 days on the period from March 1^st^ 2011 to April 10^th^ 2011. a) Cross-correlations show that sales of INC preceded sales of H. by 1 day. b) Cross-correlations show that sales of INC preceded sales of KI by 3 days. c) Cross-correlations show that sales of H. preceded sales of KI by 2 days.

To explore the timing of the purchases, we computed cross-correlations between the different sales time-series displaying an increase between February and April 2011 (all categories except iodine-based antiseptics). The cross-correlograms (Fig. 3) show that nutritional complements were purchased first, then homeopathic remedies sales peaked one day later, followed two days later by a peak of KI pills sales. No regional patterns were evidenced in the data.

## Discussion

The Fukushima crisis was a good model to study the public’s reaction to a major public health crisis. Whether the risk was real or not, we show that a part of the population chose to ignore official recommendations, seeking protection with what they perceived as preventive drugs. Table 2 allows us to hypothesize temporal links between these peaks and specific events related to the Fukushima crisis. The two main peaks of nutritional complement and homeopathic remedies sales occurred on the 18^th^ and 24^th^ of March, respectively, when a large-scale radioactive discharge was confirmed and just prior to the arrival of the radioactive cloud over France. The sequence of events, causing increasing concern, could explain the trend of purchase of different categories of products, which could be perceived of incremental efficiency: first iodine-based nutritional complements, then iodine-based homeopathic remedies, and finally KI pills.

**Table 2:** Timeline of events of the Fukushima crisis in Japan and in France from references [1], [2], [8].

Date	Japan	France
**March 11^th^, 2011**	Magnitude 9 earthquake and tsunami damage the cooling systems of reactor 1, 2, 3 of the nuclear power plant. Evacuation order to persons within 3 km.	
**March 12^th^, 2011**	Hydrogen explosion in unit 1 building. Evacuation order to persons within 10 km, then extended to 20 km.	
**March 14^th^, 2011**	Hydrogen explosion in unit 3 building.	
**March 15^th^, 2011**	Fire reported in unit 4. Hydrogen explosion in unit 2.	
**March 16^th^, 2011**	Fire reported in unit 4. Steam observed from unit 3.	
**March 17–18^th^, 2011**	First use of helicopters to dump seawater on unit 3.	
**March 20–21^st^, 2011**		French media report arrival of a radioactive cloud on March 23^rd^
**March 24^th^, 2011**		Radioactive Iodine 131 detected in center of France
**March 25^th^, 2011**		Iodine 131 and Cesium 137 above detection thresholds
**…**		
**April 25^th^, 2011**		Radioactive levels below detection thresholds.
**…**		
**December 16^th^, 2011**	All reactors officially in state of cool shutdown.	

Adverse events related to iodine consumption are limited since available studies do not reveal an association between KI and severe adverse events in the general population. Only newborns, the elderly and people with a history of thyroid dysfunction are at higher risk of developing complications [14]. However our results raise questions about behaviors that could be adopted in the context of a public health crisis. The recent influenza A/H1N1 pandemic crisis in France showed that a part of the population chose to avoid protective measures. We now show that during the Fukushima crisis a part of the population looked for its own protective measures. It is also interesting to note that, despite the absence of any official indication or biological rationale, physicians have prescribed homeopathic remedies to protect their patients’ thyroidal glands. The generally admitted absence of adverse events on account of homeopathic drugs may have played a role in physicians’ decision to prescribe them to demanding patients. This absence of adverse events could explain the rationale for such protective behavior, at no perceived cost in terms of health risk.

Our approach has some limitations: (i) Pharmacy sales are only a proxy for self-medication since we cannot quantify practices such as traditional medicines, internet drug sales, etc… (ii) The act of purchasing a drug does not imply that it has been consumed. It is possible that purchased drugs have been stockpiled in order to be available in case of a real threat. The strict regulation surrounding the delivery of KI pills favors this hypothesis, at least for KI pills. The Fukushima crisis may have acted as a reminder to people authorized to stockpile to renew their stock. But it is clear that the control surrounding KI delivery in France prevented a wider unnecessary and potentially dangerous use. In the US where KI is available without prescriptions, several media sources reported that KI manufacturers and retailers were out of stock in the days following the disaster. However no study has described the extent of the phenomena and its public health impact yet.

Habits of self-medication are deeply rooted in the population of many countries. Combined with misinformation and distrust for official recommendations, it may lead, in time of public health crisis, to dramatic consequences. In the future, public health authorities may have to adapt their reaction accordingly to account for behaviors documented in this study. Consequently, it is crucial for public health decision makers to understand how and when populations resort to self-medication in order to design more efficient public health campaigns in times of public health crisis. Our study seems to show that real-time monitoring of drug sales could be an appropriate tool in that context.

### Conclusion

Our study reports, describes, and quantifies some preventive lay behaviors during an international public health crisis. Those reactions have implications in terms of public health policy because they consist of uncontrolled use of drugs outside of their original purpose. Consequently we advocate the use of real time drug sales monitoring for instantaneous evaluation of population behavior during a global public health crisis.

## Supporting Information

Table S1 Details of groups of medication sales.
(DOCX)
