# Supplementary material for: Quantitative Assessment of Preventive Behaviors in France during the Fukushima Nuclear Crisis
Source: PLoS One. 2013 Mar 7;8(3):e58385. doi: 10.1371/journal.pone.0058385 (PMC3591339; doi:10.1371/journal.pone.0058385)
Supplement: Table S1 — Details of groups of medication sales. (DOCX) [file pone.0058385.s001.docx]

**Table S1: Details of groups of medication sales**

| **Iodine based homeopathic remedies** | BOIRON IODUM 10000K GRANULES 4G |
| --- | --- |
|  | BOIRON IODUM 1000K GLOBULES 1G |
|  | BOIRON IODUM 1000K GRANULES 4G |
|  | BOIRON IODUM 12CH GLOBULES 1G |
|  | BOIRON IODUM 12CH GRANULES 4G |
|  | BOIRON IODUM 15CH GLOBULES 1G |
|  | BOIRON IODUM 15CH GRANULES 4G |
|  | BOIRON IODUM 18CH GLOBULES 1G |
|  | BOIRON IODUM 200K GLOBULES 1G |
|  | BOIRON IODUM 200K GRANULES 4G |
|  | BOIRON IODUM 20CH GLOBULES 1G |
|  | BOIRON IODUM 24CH GLOBULES 1G |
|  | BOIRON IODUM 30CH GLOBULES 1G |
|  | BOIRON IODUM 30CH GRANULES 4G |
|  | BOIRON IODUM 3CH GLOBULES 1G |
|  | BOIRON IODUM 3CH GRANULES 4G |
|  | BOIRON IODUM 3DH GRANULES 4G |
|  | BOIRON IODUM 3DH SOLUTION BUVABLE 30ML |
|  | BOIRON IODUM 4CH AMPOULE BUVABLE 30 |
|  | BOIRON IODUM 4CH GLOBULES 1G |
|  | BOIRON IODUM 4CH GRANULES 4G |
|  | BOIRON IODUM 5CH AMPOULE BUVABLE 30 |
|  | BOIRON IODUM 5CH GLOBULES 1G |
|  | BOIRON IODUM 5CH GRANULES 4G |
|  | BOIRON IODUM 6CH GRANULES 4G |
|  | BOIRON IODUM 6DH GRANULES 4G |
|  | BOIRON IODUM 6DH SOLUTION BUVABLE 30ML |
|  | BOIRON IODUM 7CH GLOBULES 1G |
|  | BOIRON IODUM 7CH GRANULES 4G |
|  | BOIRON IODUM 8DH AMPOULE BUVABLE 12 |
|  | BOIRON IODUM 8DH GRANULES 4G |
|  | BOIRON IODUM 9CH GLOBULES 1G |
|  | BOIRON IODUM 9CH GRANULES 4G |
|  | BOIRON IODUM MHU AUTRES DILUTIONS/FORMES |
| **Iodine based nutritional complements** | GRANIONS IODE SOLUTION BUVABLE AMPOULE 2ML 10 |
|  | GRANIONS IODE SOLUTION BUVABLE AMPOULE 2ML 30 |
|  | OEMINE IODE ALGUE GELULE 60 |
|  | OLIGOBS MAXIODE GELULE 60 |
|  | OLIGOPHYTUM IODE COMPRIME SUBLINGUAL 300 |
|  | OLIGOSOL NUTRITION IODE 100MCG/2ML AMP BUV 2ML 28 |
|  | SIDN OLIGOCLASSICS IODE GELULE 50 |
| **Iodine based antiseptics** | BETADINE 10% GEL 30G |
|  | BETADINE DERMIQUE 10% SOLUT PR APPL CUTANEE 5ML 10 |
|  | BETADINE DERMIQUE 10% SOLUTION PR APPLIC LOC 125ML |
|  | BETADINE SCRUB 4% SOL PR APPL CUTANEE UNID 10ML 10 |
|  | BETADINE SCRUB 4% SOLUTION PR APPL CUTANEE 125ML |
|  | BETASEPTIC DERMIQUE 10% SOL PR APPLIC CUTANE 125ML |
|  | MYLAN POVIDONE IODEE 10% SOL PR APPL CUTANEE 125ML |
|  | MYLAN POVIDONE IODEE MOUSSANTE 4% SOL AP CUT 125ML |
|  | POLIODINE SOLUTION PR APPLICATION CUTANEE 45ML |
|  | COOPER TEINTURE IODE OFFICINALE 900ML |
|  | COOPER TEINTURE IODE OFFICINALE 1L |
|  | VIRBAC TEINTURE IODE 125ML |
|  | GIFRER TEINTURE IODE 1L |
|  | LPG TEINTURE IODE 1L |
|  | GIFRER ALCOOL IODE FAIBLE 60D 125ML |
|  | GIFRER ALCOOL IODE FAIBLE 60D 250ML |
|  | GIFRER ALCOOL IODE FAIBLE 60D 1L |
